# Effect of the Intake of Oyster Mushrooms (*Pleurotus ostreatus*) on Cardiometabolic Parameters—A Systematic Review of Clinical Trials

**DOI:** 10.3390/nu12041134

**Published:** 2020-04-17

**Authors:** Lisa Dicks, Sabine Ellinger

**Affiliations:** Faculty of Food, Nutrition and Hospitality Sciences, Hochschule Niederrhein, University of Applied Sciences, Rheydter Str. 277, 41065 Mönchengladbach, Germany; Lisa.Dicks@hs-niederrhein.de

**Keywords:** edible mushrooms, *Pleurotus ostreatus*, oyster mushrooms, cardiometabolic health, glucose metabolism, lipids, blood pressure, postprandial, chronic intake, human intervention studies

## Abstract

Cardiometabolic diseases are a leading global health challenge. Their incidence as well as progression is strongly affected by diet. Consumption of *Pleurotus ostreatus (P. ostreatus*), an edible oyster mushroom rich in functional ingredients (e.g., β-glucans), may improve glucose and lipid metabolism, blood pressure, body weight and appetite sensations. Hence, this systematic review aimed to provide an overview on the effects of *P. ostreatus* intake on cardiometabolic parameters from clinical trials, taking into account risk of bias (RoB). Relevant studies were investigated for details with consideration of the Preferred Reporting Items for Systematic Reviews and Meta-Analyses (PRISMA) guideline. The Cochrane Collaboration’s tool was used to assess the RoB. In total, eight trials included observed beneficial effects of *P. ostreatus* intake on glucose metabolism (reduction in fasting and/or 2 h postprandial glucose) and lipids (decrease in total cholesterol, LDL-cholesterol and/or triglycerides), and some found a reduction in blood pressure. In contrast, body weight did not change. Appetite sensations were not assessed. In most studies, the RoB was high or unclear due to methodological weaknesses and/or inadequate reporting. Thus, *P. ostreatus* intake may improve cardiometabolic health, but evidence for this is low. Hence, further clinical trials with an adequate study design are warranted to validate these suggestions.

## 1. Introduction

Cardiometabolic diseases such as diabetes mellitus type 2 (DMT2), dyslipidemia, hypertension and obesity are associated with increased morbidity and mortality. They are known to be the major risk factors for cardiovascular diseases (CVDs), which are still the leading cause of death in modern societies worldwide. According to the World Health Organization (WHO), 17.9 million people died from CVDs in 2016, representing 31% of all deaths [1].

Incidence as well as the progression of these cardiometabolic diseases is strongly affected by behavioral factors such as tobacco use, physical activity and diet [1]. The latter explains current interest in functional food and food ingredients which may improve cardiometabolic health [2]. At the same time, the need for sustainable food production becomes more important due to growing populations and the effects of climatic change, which represents an additional global challenge [3].

In this respect, edible mushrooms which are part of the usual diet are interesting as an alternative source of nutrients, but also for ecological reasons [4,5]. *Pleurotus ostreatus* (*P. ostreatus*), also called oyster mushroom, is one of the most cultivated edible mushrooms worldwide after white button mushroom *(Agaricus bisporus*, *A. bisporus*) [5]. *P. ostreatus* is of particular interest as it is able to colonize and degrade a large variety of lignocellulosic substrates from organic residuals and grows faster than other edible mushrooms [6]. Furthermore, *P. ostreatus* delivers various bioactive compounds such as β-glucans, which may improve cardiometabolic health [5,7]. *P. ostreatus* provides approximately twice as many β-glucans as *A. bisporus* [8], a dietary fiber that has been gaining much interest due to its beneficial role in the prevention of insulin resistance, dyslipidemia, hypertension, and obesity. The ability of β-glucans to form highly viscous solutions in the human gastrointestinal tract as well as their fermentability is ascribed to play a key role for their health-related benefits which are well known from studies with β-glucans from oats and barley [9]. β-glucans in *P. ostreatus* are side-chain/branched (1,3;1,6) polysaccharides in contrast to the linear (1,3;1,4) β-glucans in oat and barley [10]. This structural property may be beneficial for effects ascribed to an increase in the viscosity of the chymus as the β-1,6-linked glucose side chain may increase the molecule’s solubility [9]. However, β-glucans in mushrooms are partly crosslinked with chitin chains of the fungal cell wall which can reduce their solubility [11]. Other bioactive compounds which have been found in *P. ostreatus* may also improve cardiometabolic health. Mevinolin, also known as lovastatin, acts as an inhibitor of 3-hydroxy-3-methylglutaryl coenzyme A (HMG-CO_A_) reductase, and is likely to be involved in hypolipidemic effects by lowering cholesterol biosynthesis [4]. Moreover, several bioactive peptides obtained after in vitro digestion of *P. ostreatus* have been shown to inhibit the angiotensin converting enzyme [12]. This might reduce blood pressure (BP), provided that such effects would also occur in vivo. Furthermore, *P. ostreatus* is also rich in phenolic compounds. These may explain the strong antioxidant properties which were demonstrated for extracts in vitro [12].

There is an increasing number of animal studies with *P. ostreatus* which observed hypoglycemic [13,14,15], hypolipidemic [14,16], and antioxidant [17,18] effects as well as a reduction in food intake and weight gain [19]. In addition, in vitro and animal studies partly provide valuable suggestions on the underlying biological mechanisms of metabolic actions. For example, phosphorylation of AMP-activated protein kinase, gene expression of glucose transporter 4 (GLUT 4) in muscle and adipose tissue [20] and the expression of genes involved in β-oxidation increased [21]. In order to assess the efficacy of *P. ostreatus* intake on cardiometabolic health, it is necessary to systematically investigate the results from human intervention studies and to assess the quality of the studies. However, this has not been performed yet.

Thus, the aim of this systematic review was to investigate whether the intake of *P. ostreatus* modulates cardiometabolic parameters (glucose and lipid metabolism, blood pressure, body weight (BW) and appetite sensations). We reviewed clinical trials which assessed healthy subjects and subjects suffering from overweight/obesity and/or cardiometabolic diseases. We assessed the risk of bias (RoB) for each study to judge the evidence for cardiometabolic effects.

## 2. Materials and Methods

This systematic review was performed according to the Preferred Reporting Items for Systematic Reviews and Meta-Analyses (PRISMA) guidelines [22]. It was submitted to the International Prospective Register of Systematic Reviews (PROSPERO) database.

### 2.1. Literature Search Strategy

A systematic literature search was conducted in the PubMed database for human intervention studies which investigated the effect of *P. ostreatus* intake on cardiometabolic health. For the literature search, the following Medical Subject Heading (MeSH) term was used: ((“edible mushroom” OR “culinary mushroom” OR “pleurotus ostreatus” OR “oyster mushroom”) AND (glucose OR insulin OR HbA_1c_ OR “hypoglycemic effect” OR prediabetes OR “impaired glucose tolerance” OR diabetes OR cholesterol OR triglycerides OR lipids OR “lipid lowering” OR “hypolipidemic effect” OR hyperlipidemia OR “blood pressure” OR cardiometabolic OR “antiatherogenic effect” OR prehypertension OR hypertension OR atherosclerosis OR “body weight” OR appetite OR satiety OR hunger OR overweight OR obesity)). Two filters were applied: English language and human species. The database search, which was performed by one reviewer (L.D.), was finished on 31 January 2020. Eligible articles which were published up to that date were considered for inclusion.

In addition, the reference lists of the included studies were perused to identify further studies of relevance that had not been identified in the PubMed database before.

### 2.2. Inclusion and Exclusion Criteria

This review focused on intervention studies which investigated the efficacy of *P. ostreatus* intake on cardiometabolic parameters in adults. Studies were included if they investigated (1) the effect of *P. ostreatus* as a whole mushroom (fresh, cooked or dried) as well as extracts; (2) parameters of glucose metabolism (e.g., glucose, insulin, HbA_1c_), lipid metabolism (e.g., triglycerides (TGs), total cholesterol (TC), low-density lipoprotein cholesterol (LDL-C), high-density lipoprotein cholesterol (HDL-C)), cardiovascular health (e.g., systolic and diastolic BP) as well as BW and appetite sensations; (3) healthy subjects, subjects with overweight/obesity and/or cardiometabolic disorders (e.g., prediabetes, DMT2, hyperlipidemia, pre/hypertension, atherosclerosis). Studies were excluded for the following reasons: (1) treatment with other edible mushrooms than *P. ostreatus*; (2) treatment with isolated substances from *P. ostreatus* (e.g., pure β-glucans or mevinolin) or with mushroom powder or extract additionally enriched with vitamins or functional ingredients; (3) not being a clinical trial; (4) addressing other topics (e.g., anticarcinogenic effects); (5) investigating subjects suffering from severe diseases (e.g., concerning gut, liver, pancreas and kidneys, cancer), other types of diabetes than DMT2 or using medications which might favor cardiometabolic disorders (e.g., glucocorticoids, antiretroviral therapy).

### 2.3. Study Selection, Data Extraction and Risk of Bias Assessment

Two independent reviewers (L.D. and S.E.) identified relevant studies by analyzing the records using a self-made Excel template which considered the predefined eligibility criteria. First, all items were screened by title and/or abstract to exclude contributions that met exclusion criteria. Afterwards, all remaining studies were assessed for eligibility by reading the full-text article. Any discrepancies in the selection process were discussed until a consensus was reached. Finally, studies which were considered to be eligible were included in this systematic review.

Afterwards, both review authors extracted relevant data independently by using a self-made Excel template: study design, details of the intervention (amount, duration and application of *P. ostreatus*, including main ingredients based on food analysis) and of the control treatment, participants (sample size, dropout rate, demographics and baseline characteristics), outcomes (parameters on glucose and lipid metabolism, vascular function, BW, appetite sensations), times of measurement on outcome markers, and suggested mechanisms of action. Missing data were partly obtained from study authors. If available, the study registration was checked to complete data. Considering data extraction, discrepancies were also discussed by the authors.

Thereafter, both review authors independently assessed the quality of the included studies on the basis of the Cochrane risk of bias tool [23] to judge the RoB with regard to the following criteria: (1) adequate randomization method and generation of randomization list before the study (selection bias); (2) concealing treatment allocation from participants and investigators (allocation concealment); (3) blinding of participants and investigators (performance bias); (4) blinding of outcome assessment until completion of statistical evaluation (detection bias); (5) completeness of outcome data, reporting number and reasons of dropouts (attrition bias); (6) registration of study protocol, reporting full endpoints and outcomes according to registration (reporting bias). Furthermore, (7) controlled study design, details on intervention, investigation of outcome markers and on statistical evaluation, potential confounders, estimation of sample size, and comparison of baseline data were considered to assess other bias. Again, a self-made Excel template was used to check these criteria for each study. Discrepancies in the RoB assessment were also resolved through discussion.

## 3. Results

### 3.1. Study Selection and Study Characteristics

**Figure 1:**
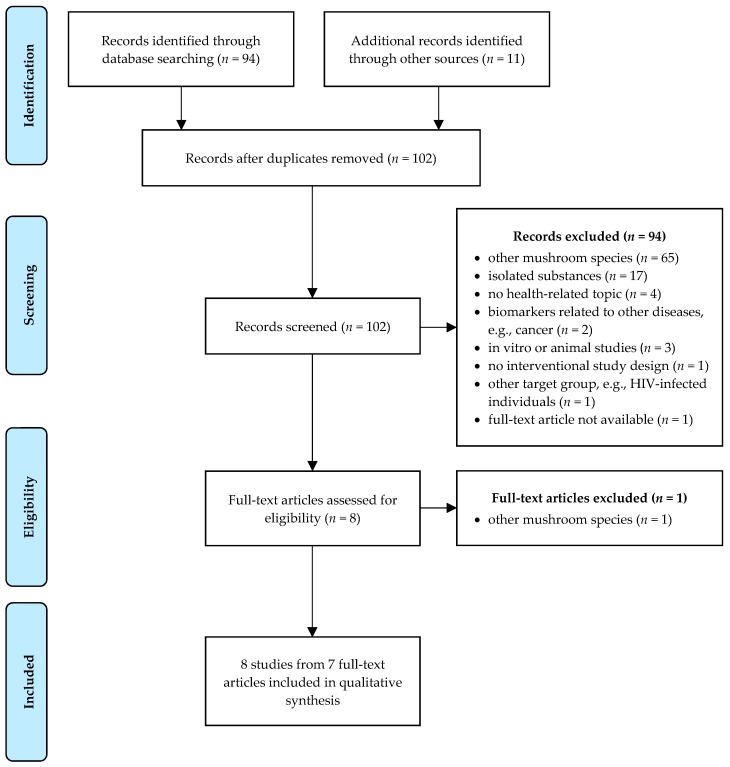
Flow-diagram of study selection process according to the Preferred Reporting Items for Systematic Reviews and Meta-Analyses (PRISMA) statement [21].

After a systematic literature search, 94 records were retrieved from the PubMed database and another 11 publications were found through other sources. After the removal of duplicates, 102 records remained. Based on titles and/or abstracts, 94 records were excluded (reasons: intervention with other mushrooms than *P. ostreatus*, *n* = 65; intervention with isolated substances from *P. ostreatus*, *n* = 17; considering other topics not related to human health, e.g., cultivation conditions, *n* = 4; considering biomarkers related to other diseases than cardiometabolic disorders, e.g., cancer, *n* = 2; in vitro or animal studies, *n* = 3; no interventional study design, *n* = 1; investigating HIV-infected individuals under antiretroviral therapy, *n* = 1; full-text article not available, *n* = 1). After reading the full-text articles, a further publication was excluded which used another *Pleurotus* spp. than *P. ostreatus* for treatment. Finally, eight studies which were presented in seven original contributions were included in the present review. A flow diagram of the identification and selection of studies is shown in Figure 1.

All studies investigated the effect of whole oyster mushrooms. These studies provided fresh [24] or cooked mushrooms [25], but in most studies, they were ingested in dried form [25,26,27,28,29,30]. All trials investigated the effect of regular, daily intake for 7–365 days [24,25,26,27,28,29,30]. Jayasuriya et al. [30] also examined the acute effect as well as the combined effect of chronic and acute intake. Most studies investigated patients suffering from DMT2 [24,25,29,30], dyslipidemia [25,26,27], hypertension [28,29] and overweight/obesity [26,28] (BMI data not shown for [24,25,29,30]). In one case, a subgroup of healthy subjects was additionally examined [30]. Outcome markers refer to glucose metabolism [24,25,29,30], lipids [24,25,26,27,28], and BP [24,25,29]. Two trials also addressed pro-/antioxidant status [26,27] and BW [25,27], whereas appetite sensations were not determined in any study. For details on study characteristics, see Table 1. Most trials were performed in Bangladesh [24,25,28,29] and further in Slovakia [26], Germany [27] and in Sri Lanka [30]. With regard to the study design, five trials which were provided in four publications were controlled [24,25,27,30]. These were performed as randomized controlled trial (RCT) [27] or as non-randomized studies, either with a two-arm parallel group design [24] or as a prospective self-controlled study (SCT) in which each participant was its own control [25,30]. In further trials including the substudy of Jayasuriya et al. on chronic *P. ostreatus* intake, a control treatment was lacking [26,28,29,30].

### 3.2. Effects of Oyster Mushroom Intake on Cardiometabolic Parameters

#### 3.2.1. Glucose Metabolism

Khatun et al. [25] provided cooked *P. ostreatus* (150 g/d, trice daily 50 g) as part of a meal in exchange for vegetables for 7 days to hospitalized patients with DMT2 or impaired fasting glucose and dyslipidemia. The plasma glucose concentration in fasting state (fasting plasma glucose, FPG) and 2 h after breakfast (2hABF) was reduced on average by 22% and 23%, respectively, after the first treatment with oyster mushrooms. After 1 week without mushrooms, FPG as well as 2hABF increased on average by 13% and 20%, respectively. A second treatment with *P. ostreatus* for 7 days again induced a mean reduction in FPG by 23% and also in 2hABF by 16%. Sayeed et al. [24] also provided oyster mushrooms (200 g/d) in exchange for vegetables, but to women with drug-treated DMT2. All investigations were performed bimonthly during an intervention for 1 year. After treatment with *P. ostreatus*, FPG decreased approximately 12% after 2 months and up to 20% after 1 year compared to baseline but did not change in the control group. Comparable changes in postprandial glucose concentrations were detectable after mushroom treatment (mean reduction approximately 14% after 2 months and approximately 28% after 1 year), but not in the control group. In the uncontrolled study of Choudhury et al. [29], men with drug-treated DMT2 ingested capsules with sun-dried powdered *P. ostreatus* (3 g/d; thrice daily amounts of 1 g) for 3 months. After intervention, FPG as well as HbA_1c_ were reduced on average by 18% and 13%, respectively. Jayasuriya et al. [30] provided lyophilized powdered *P. ostreatus* (50 mg/kg BW) as suspension to different subgroups (healthy subjects and subjects with dietetically-treated DMT2). In healthy subjects, daily intake of the mushroom suspension for 14 days reduced FPG by 6%. A control treatment was lacking. In a further experiment with the same subjects, postprandial effects were examined by using an oral glucose tolerance test (OGTT) as glucose challenge. The first OGTT was provided 2 h after ingestion of water (control), following a daily intake of *P. ostreatus* for 14 days. Thereafter, a second OGTT was performed 2 h after bolus ingestion of the mushroom suspension. The glucose concentration in plasma 2 h after OGTT was 16% lower after the intake of the mushroom suspension compared to water, which reflects the combined effect of chronic and acute intake. A further substudy with diabetics investigated the effect of a single preload with mushroom suspension which was ingested 30 min before an OGTT compared to a preload of water (control). This diminished the 2 h glucose approximately 15% and increased the 2 h insulin approximately 22%. Both treatments were performed within a self-controlled trial and were separated by 1 week washout.

#### 3.2.2. Lipid Metabolism

Khatun et al. [25] also investigated the effects on serum lipids. The decrease in FPG after the first intervention with *P. ostreatus* was accompanied by a decrease in both TGs (28%) and TC (8%). After 1 week without mushrooms, TGs increased by 11% and TC by 6%. If treatment with mushrooms was repeated, TGs and TC were again reduced by 9% and 13%, respectively. In the study of Sayeed et al. [24], the decrease in FPG after consumption of oyster mushrooms was also accompanied by a decrease in TGs and TC. This effect was already significant after 2 months (mean reduction of TGs by 19% and of TC by 12%, respectively), with a similar reduction observed after 1 year (TGs 20%, TC 20%) compared to baseline. However, LDL-C remained unchanged. No changes in TGs, TC and LDL-C were observed in the control group. Throughout intervention, TGs, TC and LDL-C were significantly lower in the mushroom than in the control group. The RCT of Schneider et al. [27] investigated adults with moderate, untreated hyperlipidemia. Daily consumption of a soup providing 30 g of lyophilized *P. ostreatus* for 3 weeks decreased TGs on average by 23%, whereas TGs increased by 25% in the control group which received a mushroom-free tomato soup instead. The changes in TGs were significantly higher in the mushroom group by 0.8 mmol/Lcompared to the control group. Significant changes in TC and LDL-C were neither detectable within each group nor between the groups. *P. ostreatus* showed significant hypolipemic effects in an uncontrolled clinical trial of Kajaba et al. [26] in which participants with primarily combined types of dyslipidemia were treated with 10 g of lyophilized powdered *P. ostreatus* daily for 6 weeks. TGs, TC and the TC/HDL-C ratio decreased significantly compared to baseline by on average 36%, 22%, and 24%, respectively. A further uncontrolled trial of Choudhury et al. [28], who provided dried powdered oyster mushrooms (3 g/d for 90 days) to hypertensive men with overweight or obesity, found a mean reduction in TC and LDL-C by 12% and 19%, respectively. In contrast to Kajaba et al. [26], changes in TGs were not observed. HDL-C was determined in all trials which investigated lipid status, but changes were not detected in any study after intervention with *P. ostreatus* [24,25,26,27,28].

#### 3.2.3. Blood Pressure

The trial of Choudhury et al. [29], already mentioned above, investigated BP in hypertensive subjects with DMT2. After a daily intake of capsules with powder from lyophilized *P. ostreatus* for 3 months, SBP and DBP decreased on average by 10% and 11%, respectively. Khatun et al. [25] also examined BP, but in normotensive subjects who received cooked oyster mushrooms daily for 7 days. Nevertheless, a significant reduction in SBP (4%) and DBP (2%) could also be observed. After consumption of vegetables instead of mushrooms for 7 days (control), DBP increased by 2%, whereas SBP remained unchanged. After retreatment with oyster mushrooms for one week, DBP decreased again by 1%, SBP remained unchanged. Sayeed et al. [24] provided fresh *P. ostreatus* to drug-treated women suffering from DMT2 (characteristics/data for BP were not provided) also in exchange for vegetables (control). BP was determined bimonthly during the one-year intervention period. Significant changes in SBP and DBP were not observed, neither within each group nor between the groups.

#### 3.2.4. Pro-/Antioxidant Status

The uncontrolled study of Kajaba et al. [26] examined the pro-/antioxidant status in adults with primarily combined dyslipidemia in addition to lipids. The concentration of conjugated dienes (CD) in plasma decreased while the activity of glutathione peroxidase (GPX) and the concentration of glutathione (GSH) in erythrocytes increased after 6 weeks of *P. ostreatus* powder intake. In contrast, superoxide dismutase (SOD) and catalase (CAT) remained unchanged. Schneider et al. [27] observed a significant decrease in oxidized LDL (oxLDL) by approximately 11% in patients with moderate hyperlipidemia after 3 weeks of treatment with oyster mushrooms, but no changes in the control group. However, the changes in oxLDL between both groups were not significantly different.

#### 3.2.5. Body Weight

BW did not change after consumption of *P. ostreatus* as well as after control treatment for 1 week [25] and 3 weeks [27].

### 3.3. Risk of Bias Assessment

**Figure 2:**
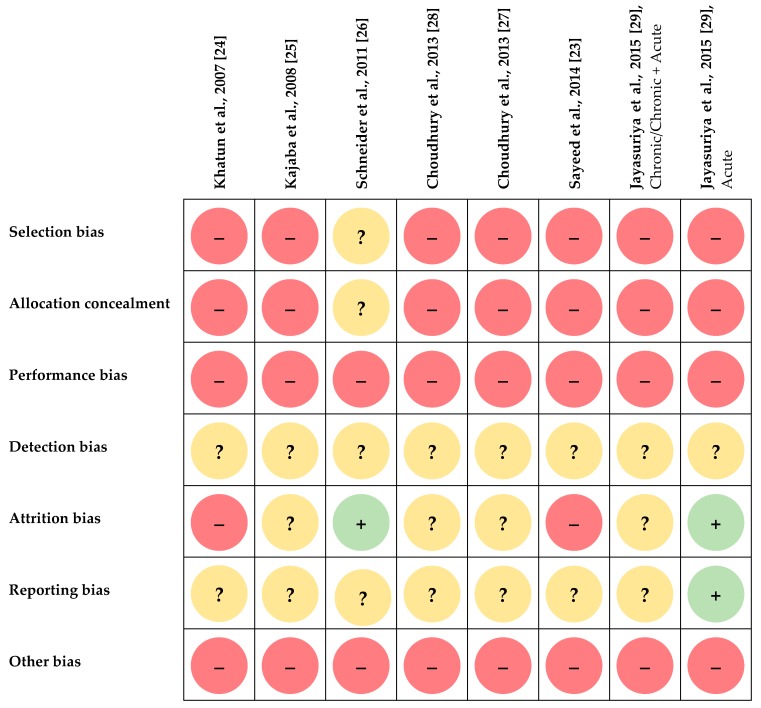
Risk of bias summary. Green (+), low risk of bias; yellow (?), unclear risk of bias; red (–), high risk of bias.

RoB was assessed for each study on the basis of qualitative criteria (Table 2). The judgements are shown in Figure 2. For all trials which were uncontrolled [26,28,29,30] or non-randomized [24,25,30], the risk of selection bias (7/8) as well as of missing allocation concealment (7/8) was rated as high. These risks remained unclear in the RCT of Schneider et al. [27], as details on the randomization procedure were lacking. Since participants were never blinded to treatment and details on blinding of investigators were never provided, the risk of performance bias was judged as high for each study (8/8). Risk of detection bias remained unclear for all studies [24,25,26,27,28,29,30] (8/8), as information on blinding of investigators during outcome assessment was not given. Except for the subtrial of Jayasuriya et al. with diabetics [30], suggestions as to the previous registration of the trial in a study register which could have revealed methodological details were missing [24,25,26,27,28,29,30]. A sample size estimation was always lacking [24,25,26,27,28,29,30]. In two studies with a dropout rate of 59% [24] and 66% [25], respectively, risk of attrition bias was high. In further studies including the subtrial with healthy subjects of Jayasuriya et al. [30], information on dropout and the intended sample size was lacking [26,28,29,30]. Risk of attrition bias was low for the trial of Schneider et al. [27] and for the substudy with diabetics by Jayasuriya et al. [30], where the number of and reasons for dropout were reported [27] or sample size was specified in the study registration [30]. Except for the latter subtrial, there were no indication concerning prospective registration of any trial in a study register [24,25,26,27,28,29,30]. Thus, risk of reporting bias could only be excluded for this subtrial [30], whereas this remained unclear for further studies [24,25,26,27,28,29,30]. The risk of other bias (8/8) was always high for several reasons: uncontrolled study design [26,28,29,30] and lack of methodological details on intervention [24,25,26,28,29,30], outcome markers (e.g., laboratory method used, period of fasting if fasting blood was collected) [24,25,30], and statistical evaluation [24]. Moreover, potential confounders were not adequately considered (e.g., source [26,27], cultivation [24,25,26,27,28,29,30] and ingredients of mushrooms [25,26,28,29,30]; check of compliance [26,27,28,29,30], nutritional behavior [24,25,26,28,29,30], physical activity [24,25,26,28,29,30], BW [26,28,29,30], and medication [24,25,26]) and might have affected the outcome. Furthermore, sample size estimation was always missing [24,25,26,27,28,29,30]. In the subtrial with diabetics, baseline comparisons were missing [30]. 

## 4. Discussion

### 4.1. Cardiometabolic Efficacy and Potential Mechanisms

To the best of our knowledge, this is the first systematic review on the effect of *P. ostreatus* on cardiometabolic parameters from clinical trials. This review considered eight trials. All studies which investigated markers of glucose metabolism found beneficial effects: these were observed after bolus consumption with regard to glucose and insulin [30], but also after regular intake of *P. ostreatus* for 7–365 days with regard to FPG [24,25,29,30] and 2 h glucose after postprandial glycemic load [24,25,30], and in one trial with regard to HbA_1c_ [29]. These effects are plausible with respect to the mechanisms ascribed to β-glucans such as delaying gastric emptying as well as slowing down subsequent carbohydrate digestion and absorption due to the increased digesta viscosity [9]. However, the observed reduction in FPG and postprandial glycemic response after an intervention period of 365 days [24] does not reflect the long-term changes in glucose control by *P. ostreatus* treatment. Whether the decrease in FPG in the study of Sayeed et al. [24] was accompanied by a decrease in HbA_1c_, as observed by Choudhury et al. [29], remains to be elucidated as HbA_1c_ was not determined. Moreover, it is not clear whether Khatun et al. [25] and Sayeed et al. [24] used a standardized breakfast as glucose challenge. Furthermore, the 2 h values in the study of Sayeed et al. [24] obtained before intervention were based on OGTT.

TGs were reduced in subjects with DMT2 in the controlled studies of Khatun et al. [25] and Sayeed et al. [24]. This may be explained by an improvement in glucose metabolism with regard to the reduction in FPG. In studies with non-diabetic subjects, TGs in fasting plasma were also diminished by treatment with *P. ostreatus* [26,27]. Only the study of Choudhury et al. [28], which was an uncontrolled study, did not observe significant changes in TGs. Of course, the reasons for the different results remain speculative, but the higher daily amount of *P. ostreatus* in studies which observed a reduction in TGs (150 g fresh mushrooms [25], 30 g powder (≙ 300 g fresh mushrooms) [27], 200 g fresh mushrooms [24], 10 g powder (≙ 100 g fresh mushrooms) [26]) compared to Choudhury et al. [28] who provided only 3 g *P. ostreatus* powder (≙ 30 g fresh mushrooms) may deliver an explanation. TC was reduced in fasting plasma in most studies [24,25,26,28] except that of Schneider et al. [27]. If LDL-C was also determined, a decrease in LDL-C was only observed in studies which found a reduction in TC [24,28], but not in the study of Schneider et al. [27]. This may be explained by the participants’ state of general health: the subjects of Schneider et al. [27] had on average a higher HDL-C (1.7 mmol/L) and a lower age (26 y) and BMI (24.0 kg/m^2^) compared to the subjects of other studies which found a reduction in TC and LDL-C (mean HDL-C: 0.9–1.2 mmol/L [25,26,28]; mean age: 43–46 y [25,26,28]; mean BMI: 27.6 kg/m^2^ [26] and 28.2 kg/m^2^ [28]; no data available for Khatun et al. [25] and Sayeed et al. [24]). The cholesterol-lowering effects by treatment with *P. ostreatus* may be mediated by β-glucans, which can increase the bile acid excretion and the activity of cholesterol 7-α-hydroxylase. In addition, short-chain fatty acids, particularly propionic acid, originating from microbial fermentation of β-glucans in the colon, can reduce de novo synthesis of cholesterol [9]. Mevinolin, a known HMG-CO_A_-reductase inhibitor, can be produced from *P ostreatus* [31], but it was not detectable in the oyster mushrooms provided by Schneider et al. [27]. Further studies did not determine mevinolin. The lack of changes in HDL-C [24,25,26,27,28] is not surprising as a meta-analysis on the impact of oat β-glucans on HDL-C could not detect any effects either [32].

All studies which investigated the effect of *P. ostreatus* treatment on BP investigated patients with DMT2. Khatun et al. [25] and Choudhury et al. [29] observed a reduction in SBP and DBP in contrast to Sayeed et al. [24]. The reasons for the different results remain unclear, as several methodological aspects (period of intervention, amount of *P. ostreatus*) which might be relevant for the results were not comparable. Moreover, the initial BP of the participants also differed (126/83 mm Hg [25]; 149/90 mm Hg [29]; Sayeed et al. [24] data not shown). β-glucans may induce antihypertensive effects by lowering insulin resistance [9], which, however, was not investigated in any study. However, an improvement in glucose metabolism was observed in all studies with regard to the results on FPG [24,25,29] and on HbA_1c_ [29].

The results on antioxidant status and lipid peroxidation are difficult to evaluate as these originate only from two studies which determined different biomarkers (oxLDL [27]; GPX, GSH, SOD, CAT, CD [26]). Furthermore, potential confounders such as smoking as well as dietary intake of antioxidants were not considered. The decrease in oxLDL [27] as well as in CD [26] after *P. ostreatus* treatment suggests a lower lipid peroxidation. However, there was no significance in the study of Schneider et al. [27] if the changes in the control group were also considered. Hence, the results of Kajaba et al. [26] have to be considered with caution due to the uncontrolled study design.

BW was only examined in two studies [25,27], but not as an outcome marker. With regard to the known effects of β-glucans, an impact of *P. ostreatus* on BW would be conceivable. Thus, investigation of BW would have been desirable in all long-term studies.

### 4.2. Consideration of Potential Confounders

In most trials, potential confounders (e.g., nutritional behavior [24,25,26,28,29,30], physical activity [24,25,26,28,29,30], BW [26,28,29,30], medication [24,25,26], and compliance [26,27,28,29,30]) were not adequately considered. Since changes in BW, lifestyle (e.g., diet, physical activity) and drug treatment may affect cardiometabolic parameters, lifestyle should be standardized to avoid confounding effects, if this is possible over the course of several weeks. Otherwise, instructions not to change diet, physical activity and drug treatment should be given before starting the intervention. Thus, the determination of BW, body composition and compliance with treatment as well as the assessment of diet, physical activity and drug treatment would be important to rule out confounders.

### 4.3. Comparability of Mushroom Treatment

The nutritional composition of oyster mushrooms is affected by the *P. ostreatus* strain used [33], but also by cultivation conditions [4], storage, drying method [34] and processing [12]. These details except that of the drying method (if *P. ostreatus* was used in dried form [26,27,28,29,30]) were never provided. This limits comparability between studies. As the β-glucan content of *P. ostreatus* differs between caps and stipes [35], the amount of β-glucans ingested by mushrooms also depends on whether the whole fruiting body or only parts of it were used for intervention. Schneider et al. [27] used the whole fruiting body, but this remains unclear in further studies [24,25,26,28,29,30].

Furthermore, the intervention periods between the trials, which investigated the effect of regular, daily *P. ostreatus* intake, differed considerably (7–365 days) [24,25,26,27,28,29,30]. Thus, the results for the same cardiometabolic outcome markers are highly difficult to compare.

### 4.4. Safety of Mushroom Treatment

Most studies examined whether treatment with *P. ostreatus* was accompanied by adverse effects. However, disorders like vomiting, diarrhea, fever and jaundice did not occur [25]. Furthermore, abnormalities in renal and liver function, which were assessed by laboratory investigations of creatinine [24,25,29,30], the glomerular filtration rate [30], alanine aminotransferase (ALT) [24,25,30], aspartate aminotransferase (AST) [30], alkaline phosphatase (ALP) [30], and gamma-glutamyltransferase (GGT) [30], were not observed.

### 4.5. Study Quality

Most trials were not robust RCTs, which are considered as a gold standard for effectiveness research, and which imply blinding, concealment of allocation, intention-to-treat analysis and a sufficiently large sample size [36]. Moreover, there was no original contribution which completely fulfilled the Consolidated Standards of Reporting Trials (CONSORT) [37]. Consequently, several aspects remain to be elucidated, as many details were not reported. This explains why the RoB of the studies which were included in the present review was mostly rated as high or unclear (Figure 2).

### 4.6. Strengths and Limitations

This review provides a systematic overview on the effect of *P. ostreatus* on cardiometabolic parameters from human intervention studies. The consideration of methodological aspects in detail as well as the RoB allows a critical evaluation of the current findings. The inclusion of studies with different target groups and with uncontrolled study designs may be considered as weakness, but a stricter selection was not possible due to the limited number of studies on this topic of research. As DMT2, dyslipidemia and hypertension are usually related to obesity and mostly have a common pathophysiological basis, similarities between the target groups may be partly assumed considering the coexistence of at least two disorders in several studies [25,26,28,29] (not clear for Schneider et al. [27], Sayeed et al. [24] and Jayasuriya et al. [30], as further data were either not investigated or not reported). Furthermore, despite the systematic approach to the literature search, it is not clear whether all studies of relevance were completely identified, as the literature search was restricted to articles which had been published in English and had been listed in the PubMed database. However, the reference list of each suitable study found in PubMed was checked to identify additional trials of relevance.

## 5. Conclusions

All studies which were included in this systematic review observed beneficial effects of *P. ostreatus* intake on glucose and lipid metabolism, and partly on BP. This might be beneficial for cardiometabolic health. However, evidence for such effects is low, as the number of studies is small and the RoB is high due to methodological shortcomings and insufficient reporting. Therefore, current findings on the efficacy of *P. ostreatus* intake on cardiometabolic parameters can only be considered as suggestions of a beneficial effect.

Thus, further clinical trials with a well-controlled study design are necessary. These should be conducted as RCTs with consideration of potential confounders. Blinding is desirable but may be a challenge when using fresh mushrooms. Powder of dried *P. ostreatus*, either encapsulated or as part of a fortified food or meal, may ensure blinding and standardization of treatment (composition, dosing) and may improve long-term compliance with mushroom intake. If the suggestions on cardiometabolic benefits were confirmed in future studies, it would be important to reveal the functional ingredients of *P. ostreatus* and to identify mechanisms of action in humans, e.g., appetite sensations, gastrointestinal hormones or insulin resistance, which plays a key role in the pathogenesis of cardiometabolic disorders.

## Figures and Tables

**Table 1:** Effect of the intake of oyster mushrooms (*Pleurotus ostreatus*) on cardiometabolic parameters—results from clinical trials.

Study, Country	Design	*n* ^a^	Participants	Interventions	IP (d)	Results ^b^			Annotations
Khatun et al., 2007 [25], Bangladesh	SCT	30 (89)	DMT2 according to OGTT or FPG >8–20 mmol/L and dyslipidemia according to NCEP ATPIII Sex (m/f): 17/13; Age: 46 ± 2 y BMI: n.a. FPG: 10.6 ± 0.7 mmol/L TGs: 2.0 ± 0.2 mmol/L TC: 4.9 ± 0.2 mmol/L HDL-C: 1.1 ± 0.1 mmol/L SBP: 126 ± 3 mm Hg DBP: 83 ± 2 mm Hg	**I_1_/I_2_**: cooked *P. ostreatus* (DAE, Dhaka, Bangladesh); 150 g/d (3 × 50 g/d), as part of the meal in exchange for vegetables **C**: no mushrooms, vegetables instead of mushrooms as part of the meal **Procedure**: I_1_ (7d) → C (7d) → I_2_ (7d)	7	**I_1/_I_2_**:	**Glucose metabolism**		Study was performed during hospital stay I_1_, I_2_, **C**: weighed, cooked and served by trained hospital staff Compliance approved by patient’s statement regarding to amount and regularity of mushroom consumption ↔ **BW** (I_1_, I_2_, C; *n* = 20)
							↓	FPG (I_1_: – 22%, *P* < 0.001; I_2_: – 23%, *P* < 0.001); 2hABF (I_1_: – 23%, *P* < 0.001; I_2_: – 16%, *P* < 0.001)	
							**Lipids**		
							↓	TGs (I_1_: – 28%, *P* < 0.001; I_2_: – 9%, *P* = 0.05); TC (I_1_: – 8%, *P* = 0.002; I_2_: – 13%, *P* < 0.001)	
							↔	HDL-C (I_1_, I_2_)	
							**Blood pressure**		
							↓	SBP (I_1_: – 4%, *P* = 0.003); DBP (I_1_: – 2%, *P* = 0.010; I_2_: – 1%, *P* = 0.017)	
							↔	SBP (I_2_)	
						**C**:	**Glucose metabolism**		
							↑	FPG (+ 13%, *P* = 0.014), 2hABF (+ 20%, *P* = 0.002)	
							**Lipids**		
							↑	TGs (+ 11%, *P* = 0.011); TC (+ 6%, *P* = 0.017)	
							↔	HDL-C	
							**Blood pressure**		
							↑	DBP (+ 2%, *P* = 0.018)	
							↔	SBP	
Kajaba et al., 2008 [26], Slovakia	UCT	57	Primarily combined types of dyslipidemia Sex (m/f): 25/32; Age: 43 y BMI: 27.6 ± 0.9 kg/m² TGs: 8.7 ± 2.2 mmol/L TC: 9.0 ± 0.8 mmol/L HDL-C: 1.2 ± 0.1 mmol/L	**I**: lyophilized powdered *P. ostreatus*; 10 g/d **C**: —	42	**I:**	**Lipids**		---
							↓	TGs (– 36%, *P* < 0.01); TC (– 22%, *P* < 0.01); TC/HDL-C (– 24%, *P* < 0.01)	
							↔	HDL-C	
							**Pro-/Antioxidant status** (subgroup, *n* = 22)		
							↓	CD (*P* < 0.02)	
							↑	GPX (*P* < 0.05); GSH (*P* < 0.05)	
							↔	SOD, CAT	
Schneider et al., 2011 [27], Germany	RCT	20 (21)	Moderate untreated hyperlipidemia (TC ≥5.2 mmol/L and/or LDL-C ≥2.6 mmol/L) Exclusion criteria: CVDs, other severe diseases (e.g., cancer, GIDs), lipid-lowering drugs, supplements (e.g., n-3 FA, phytosterols, polyglucosamine; 4 wk before and during IP), allergy to soup ingredients Sex (m/f): 9/11; Age: 26 ± 1 y BMI: 24.0 ± 1.0 kg/m^2^ TGs: 1.7 ± 0.2 mmol/L TC: 5.7 ± 0.2 mmol/L LDL-C: 3.3 ± 0.2 mmol/L HDL-C: 1.7 ± 0.1 mmol/L	**I:** lyophilized *P. ostreatus*, 30 g/d, as part of a soup; per serving (600 mL) ^c^: E, 197 kcal; P, 12.6 g; F, 8.0 g; CHO, 18.6 g; DF, 18.5 g; mevinolin, n.d. **C**: tomato soup, per serving (600 mL) ^c^: E, 274 kcal; P, 15.6 g; F, 8.6 g; CHO, 31.8 g; DF, 3.5 g; mevinolin, n.d. **Procedure**: Both soups (I, C) had to be ingested daily between 6–7 pm	21	****I**:**	**Lipids**		Blood collection after an overnight fast Subjects were advised not to change diet and physical activity throughout IP, as verified by inquiry schedules on both study days. ↔ **BW**, BMI (I, C)
							↓	TGs (– 23%, *P* = 0.015)	
							↔	TC; LDL-C; HDL-C	
							**Pro-/Antioxidant status**		
							↓	oxLDL (– 11%, *P* = 0.013)	
						**C**:	**Lipids**		
							↑	TGs (+ 25%, *P* = 0.011)	
							↔	TC; LDL-C; HDL-C	
							**Pro-/Antioxidant status**		
							↔	oxLDL	
						**TE**:	**Lipids**		
							↓	TGs (– 0.8 mmol/L, *P* < 0.001)	
							↔	TC; LDL-C; HDL-C	
							**Pro-/Antioxidant status**		
							↔	oxLDL	
Choudhury et al., 2013 [29], Bangladesh	UCT	27	Hypertension and DMT2, drug-treated, men Exclusion criteria: renal impairment, other acute or chronic diseases, addiction (except smoking) Sex (m/f): 27/0; Age: 32–68 y BMI: n.a. FPG: 10.4 ± 0.7 mmol/L HbA_1c_: 8.2 ± 0.4% SBP: 149 ± 4 mm Hg DBP: 90 ± 2 mm Hg	**I:** sun-dried (moisture 4–5%) powdered *P. ostreatus* (NAMDEC, Savar, Dhaka); 3 g/d (trice daily 2 capsules à 500 mg) **C**: —	90	**I**:	**Glucose metabolism**		Subjects were allowed to continue the medication which they were already taking.
							↓	FPG (– 18%, *P* < 0.001); HbA_1c_ (– 13%, *P* < 0.001)	
							**Blood pressure**		
							↓	SBP (– 10%, *P* < 0.001); DBP(– 11%, *P* < 0.001)	
Choudhury et al., 2013 [28], Bangladesh	UCT	14	Hypertension (SBP ≥140 mmHg and/or DBP ≥90 mmHg) and BMI >25.0 kg/m^2^, men Exclusion criteria: DMT2 (FPG ≥7 mmol/L), acute illness, malabsorption, addiction (except smoking) Sex (m/f): 14/0; Age: 44 ± 3 y BMI: 28.2 ± 0.6 kg/m^2^ TGs: 1.8 ± 0.2 mmol/L TC: 4.5 ± 0.2 mmol/L LDL-C: 2.8 ± 0.2 mmol/L HDL-C: 0.9 ± 0.1 mmol/L	**I**: electrically dried (moisture 4–5%) powdered *P. ostreatus* (NAMDEC, Savar, Dhaka); 3 g/d, as capsules (2 capsules à 0.5 g trice daily) **C**: —	90	**I**:	**Lipids**		Medication was continued if it was taken previously
							↓	TC (– 12%, *P* = 0.001); LDL-C (– 19%, *P* = 0.02)	
							↔	TGs; HDL-C	
Sayeed et al., 2014 [24], Bangladesh	CT	40 (73)	DMT2 (FPG 8–12 mmol/L), treated with OHA, age 30–50 y, BMI 22–27 kg/m^2^, women Exclusion criteria: diabetic complications, systemic illness, pregnancy Sex (m/f): 0/40; Age: n.a. BMI: n.a. FPG, HbA_1c_: n.a. TGs, TC, LDL-C, HDL-C: n.a. SBP, DBP: n.a.	**I**: fresh *P. ostreatus* (Govt. Mushroom Farm, Savar, Dhaka), 200 g/d **C**: vegetables with equivalent energy content	365 ^d^	**I**:	**Glucose metabolism** (*n* = 28)		Field workers supplied mushroom packets twice per week, checked compliance and reported the physician any irregularities in continuing mushroom consumption. Home visits also maintained to control group ↔ BMI (I, C)
							↓	FPG (– 20%, *P* < 0.001); 2hABF ^e^ (– 28%, *P* < 0.001)	
							**Lipids** (*n* = 28)		
							↓	TGs (– 20%, *P* < 0.002); TC (– 19%, *P* < 0.001)	
							↔	LDL-C; HDL-C	
							**Blood pressure** (*n* = 28)		
							↔	SBP; DBP	
						**C**:	**Glucose metabolism, lipids, blood pressure** (*n* = 12)		
							↔	FPG; 2hABF ^e^; TG; TC; LDL-C; HDL-C; SBP; DBP	
						**TE**:	**Glucose metabolism**		
							↓	FPG (*P* < 0.01); 2hABF (*P* < 0.01)	
							**Lipids**		
							↓	TGs (*P* < 0.03); TC (*P* < 0.01); LDL-C (*P* < 0.001)	
							↔	HDL-C	
							**Blood pressure**		
							↔	SBP; DBP	
Jayasuriya et al., 2015 [30], Chronic/ Chronic + Acute Sri Lanka	UCT	22	Healthy Sex (m/f): n.d.; Age: n.a. BMI: n.a. FPG: 4.5 ± 0.1 mmol/L	**I**: lyophilized powdered *P. ostreatus* (Mushroom Cultivation Centre, Export Research Board, Ratmalana, Sri Lanka); 50 mg/kg BW/d, as suspension **C**: —	14	**I**:	**Glucose metabolism**		---
							↓	FPG (– 6%, *P* < 0.05)	
	SCT			**I**: see above **C**: water **Procedure**: C, 30 min before OGTT → I (14 d) → single dose I, 30 min before OGTT	14 + acute ^f^	**TE**:	**Glucose metabolism**		OGTT: 75 g glucose, dissolved in 300 mL water
							↓	Glucose 2 h after OGTT (– 16%, *P* < 0.001)	
Jayasuriya et al., 2015 [30], Acute Sri Lanka	SCT	14	DMT2 (FPG ≥7.0 mmol/L) with dietetic treatment Exclusion criteria: DMT1, pregnancy, lactation, treated with OHA, insulin, or antihyperglycemic herbal medications Sex (m/f): n.d.; Age: n.a. BMI: n.a. FPG: n.a.	**I**: lyophilized powdered *P. ostreatus* (Mushroom Cultivation Centre, Export Research Board); 50 mg/kg BW; as suspension **C**: water **Procedure:** C, 30 min before OGTT → 1 wk washout → I, 30 min before OGTT	acute	**TE**:	**Glucose metabolism**		OGTT: 75 g glucose, dissolved in 300 mL water
							↓	Glucose 2 h after OGTT (– 15%, *P* < 0.01)	
							↑	Insulin 2 h after OGTT (+ 22%, *P* < 0.01)	

^a^ subjects completed study per protocol, in brackets: number of subjects included; ^b^ based on analysis per protocol unless indicated otherwise; ^c^ based on literature; ^d^ investigations were performed bimonthly; results refer only to values obtained after 360 days; ^e^ no information on breakfast concerning standardization; 2hABF were compared with baseline values obtained 2 h after OGTT; ^f^ bolus ingestion of *P. ostreatus* or water following a daily intake of *P. ostreatus* for 14 d; the result reflects the combined effect of acute and chronic intake. ↓ significant decrease; ↑ significant increase; ↔ no significant change (*p* > 0.05). Data on age and BMI are means ± SEMs or ranges. Values on baseline characteristics were calculated as weighted means from the data of individual groups if not provided by the authors. Missing SEMs were calculated by the SDs of individual groups. BW, body weight; C, control; CAT, catalase; CD, conjugated dienes; CHO, carbohydrates; CT, controlled trial; CVDs, cardiovascular diseases; DAE Department of Agricultural Extension; DBP, diastolic blood pressure; DF, dietary fiber; DMT1, diabetes mellitus type 1; DMT2, diabetes mellitus type 2; E, energy; f, females; F, fat; FPG, fasting plasma glucose; GIDs, gastrointestinal disorders; GPX, glutathione peroxidase; GSH, glutathione; HDL-C, high-density lipoprotein cholesterol; I, intervention; IP, intervention period; LDL-C, low-density lipoprotein cholesterol; m, males; n.a., not available; NAMDEC, National Mushroom Development and Extension Center; NCEP ATPIII, National Cholesterol Education Program Adult Treatment Panel III; n.d., not detectable; n-3 FA, omega-3 fatty acids; OGTT, oral glucose tolerance test; OHA, oral hypoglycemic agents; oxLDL, oxidized low-density lipoprotein; P, protein; *P. ostreatus*, *Pleurotus ostreatus*; RCT, randomized controlled trial; SBP, systolic blood pressure; SCT, self-controlled trial, i.e., all participants received both treatments in the same order; SOD, superoxide dismutase; TC, total cholesterol; TE, treatment effect; TGs, triglycerides; UCT, uncontrolled trial; 2hABF, 2 h after breakfast glucose.

**Table 2:** Criteria concerning the quality of the studies to assess risk of bias.

	Khatun et al., 2007 [25]	Kajaba et al., 2008 [26]	Schneider et al., 2011 [27]	Choudhury et al., 2013 [29]	Choudhury et al., 2013 [28]	Sayeed et al., 2014 [24]	Jajasuriya et al., 2015 [30], Chronic/Chronic + Acute	Jajasuriya et al., 2015 [30], Acute
**Registration of the study protocol**	?	?	?	?	?	?	?/?	√
**Study design**								
Controlled	√	✕	√	✕	✕	√	✕/√	√
Crossover	✕	–	✕	–	–	✕	–/✕	✕
Parallel group	✕	–	√	–	–	√	–/✕	✕
Self-controlled ^1^	√	–	✕	–	–	✕	–/√	√
Randomized	✕	–	√ ^2^	–	–	✕	–/✕	✕
List generated before start of the study	–	–	?	–	–	–	–/–	–
Adequate randomization method	–	–	?	–	–	–	–/–	–
Allocation concealment	–	–	?	–	–	–	–/–	–
Blinded								
Participants	✕	–	✕	–	–	✕	–/✕	✕
Investigators	?	–	?	–	–	?	–/?	?
Outcome assessments	?	?	?	?	?	?	?/?	?
**Methods**								
Details on intervention								
Source of mushrooms reported	√	✕	✕	√	√	√	√/√	√
Ingredients analyzed	✕	✕	√ ^3^	✕	✕	✕	✕/✕	✕
Cultivation conditions of mushrooms	✕	✕	✕	✕	✕	✕	✕/✕	✕
Details on the investigation of outcome markers	✕	√	√	√	√	✕	✕/✕	✕
Considering potential confounders								
Compliance	√	✕	✕	✕	✕	√	✕/✕	–
Nutritional behavior	✕	✕	√	✕	✕	✕	✕/✕	✕
Physical activity	✕	✕	√	✕	✕	✕	✕/✕	✕
Body weight/body composition	√	✕	√	✕	✕	√	✕/✕	✕
Medication	✕	✕	√	√	√	✕	?/?	√
**Statistics**								
Sample size estimation	✕	✕	✕	✕	✕	✕	✕/✕	✕
Details on statistical analysis described	√	√	√	√	√	✕	√/√	√
**Results**								
Dropout	√	?	√	?	?	√	?/?	✕
Dropouts reported	√	✕	√	✕	✕	√	✕/✕	–
Reasons reported	√	✕	√	✕	✕	✕	✕/✕	–
Baseline comparison	–	–	√	–	–	√	–/√	✕
Full endpoint/no missing data	✕	?	?	?	?	✕	?/?	√
Outcomes reported according to registration	?	?	?	?	?	?	?/?	√

✕ No, not considered; √ yes, considered; – irrelevant; ? not clear, no details available. ^1^ self-controlled trial, i.e., all participants received both treatments in the same order; ^2^ stratification by gender; ^3^ only analyzed for mevinolin and ergosterols, main ingredients of the soups according to literature.
